# The Integration of Human and Veterinary Studies for Better Understanding and Management of Crimean-Congo Haemorrhagic Fever

**DOI:** 10.3389/fimmu.2021.629636

**Published:** 2021-03-18

**Authors:** Ciaran Gilbride, Jack Saunders, Hannah Sharpe, Emmanuel Atangana Maze, Georgina Limon, Anna Barbara Ludi, Teresa Lambe, Sandra Belij-Rammerstorfer

**Affiliations:** ^1^ The Jenner Institute, Nuffield Department of Medicine, University of Oxford, Oxford, United Kingdom; ^2^ The Pirbright Institute, Woking, United Kingdom

**Keywords:** CCHF, NSDV, One Health, Hazara, vaccines, veterinary vaccines, mouse model, NHP model

## Abstract

Outbreaks that occur as a result of zoonotic spillover from an animal reservoir continue to highlight the importance of studying the disease interface between species. One Health approaches recognise the interdependence of human and animal health and the environmental interplay. Improving the understanding and prevention of zoonotic diseases may be achieved through greater consideration of these relationships, potentially leading to better health outcomes across species. In this review, special emphasis is given on the emerging and outbreak pathogen Crimean-Congo Haemorrhagic Fever virus (CCHFV) that can cause severe disease in humans. We discuss the efforts undertaken to better understand CCHF and the importance of integrating veterinary and human research for this pathogen. Furthermore, we consider the use of closely related nairoviruses to model human disease caused by CCHFV. We discuss intervention approaches with potential application for managing CCHFV spread, and how this concept may benefit both animal and human health.

## Introduction

Zoonotic diseases are caused by pathogens which circulate in vertebrate hosts and periodically spillover into human populations (1). It has been estimated that over 60% of pathogenic species in humans originally arose from animal populations (2). In the 21^st^ century alone, several zoonotic pathogens have caused epidemics such as SARS (3) and MERS coronaviruses (4), avian influenza (5), and Ebolavirus (6, 7), as well as pandemics of swine flu (8) and the newly emerged SARS-CoV-2 (9), which alone has caused an estimated 2.25 million deaths up to February 2021 (10).

While it is difficult to estimate the continuous economic burden of zoonotic disease, localised epidemics and worldwide pandemics can induce instant and deep economic shock which has important secondary impacts on global health outcomes. The 2015 MERS outbreak in South Korea infected 186 confirmed individuals and caused a total of 36 deaths, with the overall economic damage to South Korea approximated at USD$8.5 billion (0.6% GDP) (11, 12). Estimates for the larger 2002-2003 SARS epidemic place the economic costs at $3.7 billion (2.6% GDP) in the Hong Kong epicentre (13, 14), while the recent COVID-19 pandemic economic impact may be greater with estimates for the second quarter alone of 2020 showing a 2% fall in GDP worldwide (15).

Economic damage can directly cause human mortality, particularly in low- or middle-income countries where the healthcare systems may be less robust. The 2014-2016 Ebolavirus epidemic resulted in approximately 11,000 direct deaths (7), but the subsequent overwhelming of the healthcare systems in Guinea, Sierra Leone, and Liberia caused an estimated 10,000 further deaths (16). Avian influenza virus strains can cause fatal disease in humans and introduction into Indonesia in 2009 caused devastation to livelihoods as a result of culling 11 million poultry. The subsequent closure of 30% of the country’s farms created severe disparities in education and nutrition in the worst affected communities (17).

Human activities, such as accelerated deforestation, encroachment into natural animal habitats, or climate change (18), exponentially increase the likelihood of exposure and spillover of novel zoonotic pathogens (19). Due to their potential impact to human health, the WHO and other healthcare agencies have identified pathogens of concern with epidemic or pandemic potential (**Table 1**). The list represents zoonotic diseases that have caused or may lead to outbreaks, and many lack effective and timely control measures that are crucial for preserving human and animal health.

**Table 1:** WHO Blueprint Diseases and their principal mammalian reservoirs.

WHO Blueprint Disease (20)	Mammalian reservoir(s)
COVID-19 (recently added)	Fruit bats (speculative) (21), pangolin (speculative) (22)
Crimean-Congo Haemorrhagic Fever (CCHF)	Cattle (23), goats (24), sheep (23), camels (25), horses (26), donkeys (27)
Ebola virus disease	Fruit bats (28)
Marburg virus disease	Fruit bats (29)
Lassa fever	Multimammate mouse (*Mastomys natalensis*) *(* 30)
MERS coronavirus disease	Bats, alpacas, camels (31)
SARS coronavirus disease	Horseshoe bats, palm civets (32)
Nipah virus disease	Flying foxes (33), pigs (33)
Rift Valley Fever	Sheep, goats, cattle (34)
Zika	Rhesus monkeys, sheep, goats, cows, horses, bats, carabaos, orangutans (35)
Disease X (future disease outbreak of unknown origin)	Unknown

The One Health approach acknowledges the interdependent relationship between human and animal health, together with our shared environment. The need to increasingly consider all parts of the ecosystem arose as a result of global concerns including antimicrobial resistance and the prevalence of emerging infectious diseases transmitted between animals and humans (36). One Health approaches aim to address such issues using control measures that are ultimately rooted in achieving better veterinary and human health outcomes. Control measures can include detailed epidemiology facilitating identification of transmission routes, disease surveillance, or more overt strategies such as the development of therapeutic and preventative healthcare measures (37). A conceptual framework encompassing a One Health strategy requires concerted inter-disciplinary efforts from many professions including social science, healthcare and epidemiology. This level of collaboration is necessary to scale solutions from local to national to global levels to help manage the spread of zoonotic diseases (38).

An identified zoonotic disease of concern, Crimean-Congo Haemorrhagic Fever (CCHF), is a tick-borne disease caused by CCHFV, which affects humans and can cause severe haemorrhagic symptoms with fatal outcomes (39). As noted in **Table 1**, numerous vertebrates can act as reservoirs of CCHFV to help maintain the virus. Reservoir animals can experience transient viremia and may develop antibody responses towards CCHFV, but no clinical disease is observed (40). As such, further work is needed to identify the main drivers of disease underpinning pathogen transmission.

In this review we discuss the value of integrating veterinary and human research in the field of CCHF by discussing findings from recently developed animal models and underlining the benefit of exploring closely related nairoviruses such as Nairobi Sheep Disease Virus (NSDV) and Hazara Virus (HAZV). In addition, we address current vaccine candidates for preventing CCHF and a range of approaches that could be key aspects to One Health approaches to combat CCHF.

## Crimean-Congo Haemorrhagic Fever

The control and management of zoonotic diseases, such as CCHF, relies heavily on knowledge of the disease. Frequently the causes and impacts of zoonoses are complex and poorly understood (41). Animal reservoirs of CCHFV encompass domestic ungulates, while small mammal and bird populations are thought to play a role in immature tick maintenance and CCHFV transmission (42, 43). For a successful One Health approach, it is crucial that sufficient understanding is acquired on animal reservoirs, amplifying hosts and transmission patterns to adequately understand both human and animal disease, while considering CCHFV maintenance and circulation in ticks (44). Attainment of greater knowledge in these areas will support the implementation of control measures, such as prophylactic vaccines for human or animal use (45).

CCHFV is maintained through a tick-vertebrate-tick transmission cycle (46, 47). Ticks require a blood feed on small vertebrates to progress in their life cycle from larvae to nymph, and then feeding on large mammals, such as cattle and sheep, to progress from nymph to adult tick (47). Though their role is not fully understood, flighted birds can carry ticks and are thought to contribute to the expansion of tick populations to other territories (43, 48, 49). CCHFV is contracted through two major routes. The first is through direct contact with infected ticks, either through the bite of an infected tick (44) or tick pulverisation on open wounds exposing an individual to the virus (50). The second transmission route is contact through an open wound with the blood or bodily fluids of an infected person or animal. High risk occupations for CCHFV infection include veterinarians, farmers, and abattoir workers in endemic areas that are in close proximity to livestock (50–52). Human-to-human transmission can occur following close contact with infected individuals, posing considerable risk for nosocomial outbreaks (53–55).

CCHF is one of the most widespread tick-borne diseases (42). Cases in humans are frequently reported in countries across Asia, Africa and Europe (39) and CCHFV is increasingly being identified in new geographical regions (56, 57). Human infections can range in severity from subclinical or mild disease with non-specific symptoms, to severe disease which can cause fatal haemorrhagic disease (54). The mortality rate during annual outbreaks is estimated to be between 5-30% (39, 46). There are potential differences in CCHF severity between CCHFV endemic geographical regions and it is thought that many human CCHF cases remain subclinical which affect calculated mortality rates (58). A sero-epidemiological survey in Turkey estimated that 88% of CCHFV infections were subclinical but it is unclear if similar asymptomatic rates occur in other endemic countries (59).

Controlling and curbing CCHFV circulation represents a public health priority, especially in countries where the virus is endemic. Better understanding of disease is needed to accelerate therapeutic and interventional treatments. Animal models are critically important in underpinning our knowledge of disease dynamics and progression. As discussed in the following section, understanding of CCHF can be supported through the study of animal models and closely related nairoviruses.

### Animal Models of CCHFV Infection

CCHFV is typically studied in BSL-4 conditions due to the high risk of severe or fatal haemorrhagic disease in humans, as at present there are no therapeutics or vaccines available (60). Mouse and non-human primate (NHP) models exist for studying CCHFV pathogenesis in these high containment settings. There are a limited number of CCHFV mouse models due to the need to supress or pre-empt the immune system, as infection of immunocompetent mice with CCHFV does not result in overt disease (61). Historically, the first model involved new-born mice, but it was considered inadequate as pathogenesis in new-born mice involved severe damage to the central nervous system (46), a pathology not representative of human disease. Typical murine models involve knock-out of IFN-I receptor (61, 62) or STAT1 (63) targeting the type-I interferon (IFN-I) response. The same phenotype can be induced in immunocompetent mice by treatment with antibodies against IFN-1 that block IFN-1 signalling prior to CCHFV infection (64). Such knock-out models have been useful, as some of the main clinical symptoms and disease pathogenesis of CCHF manifest, particularly infection of the liver and spleen, within 4-5 days of inoculation (61–63). These animal models have been informative in determining viral tropism and have been used to assess vaccine candidates (65, 66).

Development of NHP models of infection are often considered a pinnacle of replicating human infections in an animal model. However, disease pathology can differ across NHP species for many emerging pathogens, requiring infection studies on a breadth of species to determine a suitable model. For CCHFV, cynomolgus macaques are a suitable model of infection mimicking human disease (67). In cynomolgus macaques, disease pathology appears to be similar to that of humans, with all infected animals developing mild to severe disease, and a proportion of infections displaying a lethal outcome (67). A larger immunocompetent animal model of infection, such as a NHP model, is generally deemed crucial to assess vaccines and therapeutics against CCHFV infection, however, they are more expensive than small animal knock-out models.

### CCHFV Analogues for Understanding *Orthonairovirus* Pathogenicity

In order to overcome the difficulties of studying pathogens that cause severe disease in humans, researchers frequently use animal–specific pathogens that have a close phylogenetic relationship and pathology profile to those that cause the disease of interest in humans (68).

NSDV, known as Ganjam virus in India, is an *Orthonairovirus*, closely related to CCHFV (69). NSDV causes Nairobi Sheep Disease (NSD), first described in Kenya during a 1917 outbreak of severe haemorrhagic gastroenteritis in sheep that were relocated from a NSDV-free area to one where NSDV circulated (70). Since then, outbreaks of NSDV have been observed infrequently in sheep and goats.

Like CCHFV, NSDV is also transmitted by ticks. The main symptoms of small ruminant NSDV infection are a febrile illness with diarrhoea, followed by haemorrhages with mortality rates up to 90% (71–73). However, NSDV has a low level of zoonotic potential with evidence of human infection rarely documented (74, 75).

There are a number of analogues between NSDV infection of sheep and CCHFV infection of humans, represented in **Table 2**. Individuals and animals generally undergo a febrile illness, followed by a haemorrhagic phase in the same organs, predominantly the gastrointestinal tract; both produce leukopenia and injury of the liver and spleen (85). Infection of susceptible hosts with NSDV or CCHFV induces a similar pro-inflammatory immune reaction (71, 86) as well as a long-lasting antibody response (52, 87). NSDV infects more organs than CCHFV, and death from NSDV typically occurs within 10 days post infection (72, 73), compared to 5-14 days following onset of illness in CCHFV infection (46).

**Table 2:** Features of Crimean-Congo Haemorrhagic Fever Virus and Nairobi Sheep Disease virus.

Feature	CCHFV	NSDV
Virus structure	Enveloped negative sense ssRNA virus with tripartite genome (76)	Enveloped negative sense ssRNA virus with tripartite genome (76)
S segment amino acid sequence similarity	62-63% (77)	
Vector	Ixoxid ticks, predominantly *Hyalomma* genus (78)	Ixoxid ticks, predominantly *Rhipicephalus* and *Haemophilus* genus (69)
Clinical Disease	Humans (79)	Sheep and goats (69)
Mortality	5-30% (46)	90% (80)
Symptoms in susceptible host	Fever, myalgia, headache, nausea, soft tissue haemorrhage, epistaxis, hematemesis (81)	Fever, diarrhoea, gastro-intestinal haemorrhage, soft tissue haemorrhage (71)
Tissue pathology	Isolated in lung, liver, and spleen (82, 83)	Isolated in lung, liver, spleen, and intestines (84)

The major advantage of using NSDV infection in small ruminants as a model for CCHFV infection over the current mouse models is the ability to study lethal *Orthonairovirus* infection in an immuno-competent organism. The limitation of NSDV is that the virus is of a different species to CCHFV, with a different host tropism. However, the close phylogenetic relationship between NSDV and CCHFV increases the chance that results may be translatable, and the similarities in haemorrhagic pathogenesis suggest that NSDV infection can be used to investigate systemic and immunological effects of *Orthonairovirus* infection, at a lower risk to human health compared to using NHP models of CCHFV.

HAZV is another nairovirus that may be used as a model of CCHFV infection. Discovered in Pakistan in *Ixoxid* ticks (88), HAZV can be handled in BSL-2 facilities and is non-pathogenic to humans, unlike CCHFV (88, 89). Significantly, HAZV is considered the virus most closely related to CCHFV; HAZV is in the same sero- (90) and genogroup (76) as CCHFV and there is a high level of structural homology between the nucleoprotein of CCHFV and HAZV (91, 92).

Researchers have used HAZV infection for pre-clinical investigations, such as *in vitro* assays to assess the effects of the therapeutic agent ribavirin in combination with other treatments at preventing HAZV infection (93). Ribavirin is commonly prescribed for treating CCHFV (94) and therapeutics which show efficacy against HAZV have been speculated to be effective against CCHFV (93). Parallels between the pathology of CCHFV and HAZV infection have also been observed in immunocompromised mouse models. Infection of IFN-1 knock-out mice with HAZV resulted in lethal disease in all mice (95). The pathology, mortality, and clinical signs were highly similar to those induced by CCHFV infection in the same immunocompromised mouse strains (96). In contrast, wild-type mice were not susceptible to HAZV or CCHFV infection (95).

The close similarity of HAZV with CCHFV has also led to the suggestion to use HAZV to investigate nairoviral infections in the amplifying hosts of CCHFV, namely sheep, goats, and cattle (97). Experimental challenge of domestic animals, including sheep and cattle, with HAZV does not cause symptomatic disease, and HAZV replication was also not observed in sheep or cattle during a recent challenge study (97). Viremia has been observed upon HAZV challenge in a number of other animals including rhesus monkeys and donkeys (98).

As HAZV does not cause overt disease in human or animals, unlike CCHFV and NSDV respectively, HAZV cannot be used to investigate the haemorrhagic pathogenesis observed in human CCHFV infection. A second limitation of HAZV as a model for CCHFV is the lack of natural infection reported in domestic animals and an unknown host species, which limits corollaries being drawn regarding transmission modes or model studies.

Due to the high levels of homology between HAZV and CCHFV, HAZV could provide a viable model virus to study the molecular biology and pathogenicity of CCHFV. HAZV may also be used for the screening of preventative or therapeutic measures that could be translatable for CCHFV, with the use of HAZV precluding the need for experimentation in BSL-4 containment.

## Controlling Zoonotic Transmission of CCHFV

Despite the presence of CCHFV across a large geographical range and the severity of outbreaks, it is difficult to estimate the disease burden due to the low level of active CCHF surveillance and reliance on passive surveillance with high levels of under-reporting. There is also limited diagnostic capability in many endemic regions, with the uncertain frequency of subclinical infections adding to this issue. As a result, it has been suggested that the burden of CCHF disease is greater than estimated from official case reports (99, 100). Asymptomatic infections may be common and mild disease can present as non-specific febrile symptoms (101). The awareness of CCHF disease and symptoms is low among physicians and veterinarians even in endemic countries (102), and increasing the awareness of CCHF clinical symptoms among physicians and veterinarians is the first step towards improving access to diagnosis for mild cases, and to prevent nosocomial outbreaks (103).

Implementation of better diagnostic frameworks to improve surveillance strategies for CCHFV infections is an important aspect for a comprehensive One Health approach for disease management (**Table 3**). A key part of this strategy is the use of rapid laboratory diagnosis for humans by reverse transcription polymerase chain reaction (RT-PCR), a highly sensitive tool to detect CCHFV RNA in individuals (104). Crucially, there are not always laboratory facilities equipped to perform PCR and diagnose CCHF in less developed rural areas, or they may lack the capacity needed during outbreaks (105). This can result in distant reference laboratories being relied upon, delaying diagnosis (100). The lack of specific clinical presentation also adds difficulty to seeking diagnosis. Improved diagnostic capacity and accessibility in both clinical and field-based settings by the establishment or improvement of regional laboratories in endemic areas would allow earlier detection of positive cases and pre-emptive interventions to be undertaken (106). Increased clinical diagnosis would also identify more asymptomatic and mild cases and improve the estimation of CCHF disease burden.

**Table 3:** The different control measures that may facilitate a CCHF One Health approach and the current status or challenges for their implementation.

Control measure strategy	Current status
Immunisation of humans	Limited licensure of one inactivated vaccine in Eastern Europe Multiple vaccine candidates show promise in pre-clinical studies No published assessment of candidates in human trials
Immunisation of animals	Multiple vaccine candidates show promise in pre-clinical studies Lack of disease burden means a lack of economic incentive to vaccinate
Tick control	Acaricidal agents known to be effective at reducing vector-borne disease rates Environmental implications and logistical issues of widespread usage
Diagnostic, education, and surveillance	PCR available but often limited in capacity or inaccessible Sero-surveillance rates increasing (44)

Active CCHF surveillance is also vital, as has been displayed by the increasing frequency of serological studies to detect anti-CCHFV antibodies in human populations (99, 107). The data from these sero-surveillance investigations have provided a key insight into the seroprevalence of populations and can be used to evaluate potential risk of exposure in endemic areas (44). Interpretation of such studies should be considered with caution as is that the serological methodologies are often inconsistent and accuracy is not always quantified (58). Additionally, seroprevalence measurement does not resolve the missing CCHF knowledge regarding incidence of subclinical infections versus symptomatic disease. Serological studies in both domestic and wild animal species, as well as surveying distribution and viral presence in tick species, should also be integrated into CCHF surveillance strategies (47, 108). Assessing CCHFV in the amplifying and natural hosts can increase the understanding of the geographical spread of CCHFV and provide estimates of circulating viral quantities, though there is additional complexity to animal and tick surveillance (99, 109). Establishment of CCHF surveillance programmes could determine potential levels of risk to human populations in endemic areas by monitoring the prevalence of virus in humans, animals, or tick vectors and this may identify where disease management interventions could be most useful.

CCHF impacts human health, with hundreds of cases officially reported each year and an obvious need for improved control measures including preventative vaccinations or therapeutic treatments (**Table 3**). There are currently no CCHFV vaccines licenced for widespread usage (110), although one has been licenced in Eastern Europe which uses an inactivated virus platform (111). Studies investigating the immune response to this formalin inactivated vaccine have demonstrated that after four doses, low levels of neutralising antibodies are induced (112). Multiple other vaccine candidates for CCHFV have been developed using a wide range of platforms to deliver vaccine antigens (113–116). For example, two doses of a protein-based subunit CCHFV vaccine that targeted Gn and Gc of the glycoprotein precursor achieved neutralising antibody responses, but did not confer protection in mouse challenge models (117). A DNA vaccine expressing the CCHFV full-length glycoprotein induced humoral and cellular immunity and was protective after two doses in mouse challenge models (118). Viral vectored vaccines that used the MVA vector to deliver full length glycoproteins as immunogens against CCHFV also induced humoral and cellular responses; the vaccine was 100% protective against CCHFV in immunocompromised mouse models (119). Efforts are now needed to test and then translate putative vaccine candidates into veterinary and human vaccines which can protect against CCHFV (120). It is likely that these medicinal interventions would be stockpiled in endemic countries and used in outbreak situations as has been suggested for other outbreak pathogens such as Nipah or MERS coronavirus (121).

While immunising livestock and poultry against infectious pathogens is an economically important practise to avoid disruption to the food and textiles industry (122), vaccination of farmed animals against diseases can also reduce the likelihood that zoonotic pathogens will be transmitted to humans (123, 124). There are, however, barriers to the development of effective CCHFV vaccines for use in veterinary settings (**Table 3**). There are no economic incentives for farmers, livestock producers or agri-industry to vaccinate against CCHFV. Without an appreciable level of disease in animal hosts, and the accompanying loss of income to those working with animals, the incentive to vaccinate animal reservoirs purely for the benefit of human health may be limited. As such, outside investment may be needed to incentivise this approach. Alternatively combining vaccines that combat impactful veterinary pathogens with a vaccine against CCHFV may persuade key stakeholders to implement vaccination regimens.

Further methods may be considered to manage CCHFV in animal reservoirs. Targeting the disease vector is common in reducing arbovirus transmission (125) and therefore reservoir livestock can be treated with acaricidal agents to remove ticks (**Table 3**) (126). Unlike with vaccination against CCHFV, treatment of ticks offers direct benefits to animal health (127). However, the acaricidal agents can contaminate animal products (128), and annual deaths from organophosphates greatly outnumber those from CCHFV (126). Due to the environmental implications of acaricide usage (129), it would not be feasible to apply acaricides across the large areas needed to supress wild tick populations. Furthermore, the eradication of ticks across large regions of lands would negatively affect the ecosystem, causing unspecified environmental damage (130). While likely to be insufficient alone, careful control of ticks in livestock would be a valuable tool alongside vaccination to reduce CCHFV infections in humans.

## Conclusion

To mitigate the risk and impact posed by CCHF it is vital that sufficient knowledge on human infection and the interplay with the animal reservoir is delineated. Though limited in the past, the increasing availability of animal models has supported the study of CCHF as a disease and the causative agent CCHFV. These models are already playing a significant role assessing the preclinical efficacy of CCHF vaccines. Similarly, the study of closely related nairoviruses such as NSDV and HAZV that are non-pathogenic to humans can further advance our understanding of CCHFV, due to the similarities of the virus infection and subsequent disease. These closely related pathogens can represent valuable models for CCHFV infection, though all research must be viewed with the caveat that there are differences between CCHFV and the model pathogens which must be taken into account.

CCHF has historically been overlooked as a disease of impact due to being largely under reported in humans and the asymptomatic nature of CCHFV in animal reservoirs that enables the virus to circulate undetected. There is little incentive for treatment of animals or surveillance until zoonotic transmission occurs. Without co-ordinated rapid diagnostic testing in tandem with sero-surveillance mechanisms, the first evidence of circulation is frequently after zoonotic transmission, with those working closely with animals put at considerable risk with no foreknowledge (50).

Given the high mortality rates seen during sporadic (but now almost annual) outbreaks of CCHF, better management approaches are essential in countries where CCHFV is endemic and significant health risks exist. This is urgently needed due to the potential for increased incidence of CCHF cases and growing geographical distribution of *Hyalomma* ticks resulting from environmental changes. There are obvious challenges to alleviating the threat of CCHFV, but ultimately, implementation of a One Health approach to CCHFV management and control while focusing on integrating human and veterinary studies would be of huge benefit to human health.

## Author Contributions

CG, JS and EA contributed to writing of the original draft. CG, JS, HS, EA, GL, AL, TL and SB-R contributed to review and editing to produce the final manuscript. All authors contributed to the article and approved the submitted version.

## Funding

This research was funded by the Biotechnology and Biological Sciences Research Council (BBSRC) [BB/R019991/1] and the National Institute of Health Research (NIHR) [16/107/06].

## Conflict of Interest

The authors declare that the research was conducted in the absence of any commercial or financial relationships that could be construed as a potential conflict of interest.
